# Exposure to Adverse Childhood Experiences and Mental Health Issues in a Young‐Adult Sample of University Students in Bangladesh: A Cross‐Sectional Study

**DOI:** 10.1002/hsr2.70712

**Published:** 2025-04-18

**Authors:** Shamima Akter, Raufun Hasan Arnob, Md. Ashik Ulla Ashik, Md. Mosfequr Rahman

**Affiliations:** ^1^ Department of Population Science and Human Resource Development University of Rajshahi Rajshahi Bangladesh; ^2^ Institute for Population and Social Research Mahidol University Salaya Thailand

**Keywords:** adverse childhood experiences, anxiety, Bangladesh, depression, mental health, university, young adult

## Abstract

**Background and Aims:**

The prevalence of adverse childhood experiences (ACEs) is remarkably high in Bangladesh, and there is well‐documented evidence establishing a relationship between ACEs and mental disorders in children and adults. However, little is known about how ACE exposure affects young adults' mental health. Therefore, this study aims to estimate the prevalence of ACEs and assess the relationship between ACE exposure and developing anxiety and depression symptoms in a young adult sample of university students.

**Methods:**

A cross‐sectional survey comprising 858 young adult students aged 18–29 years, enrolled in an undergraduate or graduate level of study at a large university in Bangladesh, was conducted between October and December 2023. ACE exposure was measured using ten items from the CDC‐developed ACE tool. Self‐reported anxiety and depression symptoms were assessed using the Generalized Anxiety Disorder 7‐item Scale and the Patient Health Questionnaire‐9. The associations between the variables of interest were assessed using multivariable logistic regression.

**Results:**

More than half (54.1%) of the students reported experiencing ACEs. The prevalence of developing moderate‐to‐severe anxiety and depression symptoms was 34.4% and 71.6%, respectively. One item increase in the ACE score increased the odds of experiencing moderate‐to‐severe anxiety symptoms by 27% (adjusted odds ratio [AOR]: 1.27; 95% confidence interval [CI]: 1.16–1.38) and moderate‐to‐severe depression symptoms by 19% (AOR: 1.19; 95% CI: 1.08–1.31).

**Conclusion:**

ACE exposure is prevalent in this sample of university students and is associated with developing anxiety and depression symptoms. Exposure to ACEs should be considered in developing intervention strategies for improving young adult students' mental health.

## 1 Introduction

The increasing incidence of mental problems in young individuals has emerged as a significant and critical global public health concern. The majority of mental illnesses start in youth, even though they are often first discovered later in life. According to the World Health Organization (WHO), around 50% of mental disorders begin before the age of 14, and 75% begin before the age of 24 [1, 2]. Prevalence rates for the majority of mental health disorders are higher among adolescents and young adults than in other stages of life, with a prevalence of 14% and 13.6% for the age groups 15–19 years and 20–24 years, respectively [3]. Research indicates that the prevalence of moderate‐to‐severe anxiety and depression symptoms in young adult university students in Bangladesh is 25.1% and 39.4%, respectively [4]. Such a high proportion poses great concern as mental disorders have been associated with various negative consequences, including hindering the ability of young individuals to reach their full potential [5, 6], restricting their access to mental [7] and physical [8, 9] healthcare, subjecting them to inadequate education and limited occupational opportunities [10], exposing them to stigma, social isolation, discrimination, and human rights violations [11, 12], and reducing their life expectancy [13]. This proportion emphasizes the necessity of identifying interventions that are designed to mitigate risk factors and improve protective factors associated with mental health disorders. To make significant progress in this area, we must target the leading causes of mental disorders, such as adverse childhood experiences (ACEs), to reduce disease prevalence and burden [14].

ACEs—unfavorable experiences that a child may encounter in the first 18 years of life—are a serious global public health issue [15] and have a negative impact on physical and mental health outcomes and well‐being throughout life [16, 17]. ACEs are classified as instances of abuse (including physical, sexual, and emotional abuse), home difficulties (such as violence, mental illness, parental separation, and the presence of criminal household members), and neglect (either physical neglect, emotional neglect, or both) [18]. According to estimates from the WHO, 1 billion children between the ages of 2 and 17 experience physical, sexual, or emotional abuse each year, contributing to the tragically high global burden of childhood trauma [19]. A review of 42 studies across the globe documented that a significant proportion of young adults, ranging from 22% to 87%, have encountered at least one form of adverse experience [20]. Another recent meta‐analysis that includes 206 papers from 22 countries, with 546,458 adult participants, reported that the pooled prevalence of five levels of ACEs was 39.9% for no ACE, 22.4% for one ACE, 13% for two ACEs, 8.7% for three ACEs, and 16.1% for four or more ACEs [21]. However, ACEs are more common in low‐ and middle‐income nations like Bangladesh, where 98% (1394 out of 1416) of children reported experiencing lifetime psychological abuse at home, and an astounding 99% (1398 out of 1416) of children surveyed experienced physical abuse in their lifetime [22, 23]. These numbers are a significant cause for concern. In 2015, every member state of the United Nations embraced the 2030 Sustainable Development Goals (SDGs), which serve as a call to action aimed at eradicating poverty and inequality, safeguarding the planet, and guaranteeing that all individuals have access to health, justice, and prosperity. The SDGs consist of 17 goals, encompassing a total of 169 specific targets. Nonetheless, SDG 16.2 seeks to eradicate violence against children. Because of this, the high rate of ACEs found in many studies raises considerable concern for countries globally, including Bangladesh, where the government demonstrates a strong commitment to fulfilling the targets of the SDGs.

The link between ACEs and mental diseases is of significant interest to the field of public health due to the substantial impact and consequences of mental illness. Arnett [24], in his theory of emerging adulthood, delineated the age range of 18 to 25 years as a novel life stage and characterized the salient attributes of young people. This phase is marked by identity exploration, instability, self‐focus, a sense of liminality (neither adolescent nor adult), and potentialities [24]. Considering these developmental traits and distinct requirements of young people, outcomes that may be less pertinent to older adults could hold significant importance during young adulthood. Prior studies have revealed an association between childhood adversity and an increased likelihood of experiencing anxiety and depression symptoms during young adulthood [25, 26, 27, 28, 29, 30]. There is a scarcity of literature regarding the correlation between ACEs and mental health problems among young adults in Bangladesh. Research utilizing nationally representative data from the global school‐based student health survey among school‐going adolescents aged 11 to 17 years has found that exposure to physical or sexual violence throughout childhood is associated with suicide behavior, depressive symptoms, and anxiety [31, 32]. A recent study of young‐adult male child sexual abuse survivors aged 18–23 years living in urban and rural areas in Bangladesh found that suicide and depression were significantly associated with higher odds among those who disclosed sexual abuse, experienced repetitive abuse, and were physically abused during instances of sexual assault [33]. A further study among 1844 students from the largest university in Bangladesh found that students who had a familial history of psychiatric disorders were more likely to have signs of depression [34]. Nevertheless, this study lacked the utilization of scientifically rigorous instruments to assess ACEs. The growing incidence of mental diseases as well as ACEs emphasizes the need for more thorough research into the association between ACEs and mental diseases among young adults, who make up about one‐fifth of the whole Bangladesh population. Therefore, this study aims to assess the prevalence of ACEs and their relationship with developing anxiety and depression among a young‐adult sample of university students in Bangladesh. We hypothesize that experiencing ACEs develops the symptoms of anxiety and depression among young adults. The results of this study will help university administrators and policymakers develop an effective intervention plan to enhance the mental well‐being of young people in Bangladesh.

## 2 Methods

### 2.1 Participants and Procedure

A cross‐sectional paper‐based survey was done between October and December 2023 among students enrolled at the University of Rajshahi in Bangladesh. The University of Rajshahi, with over 33,000 students, is the second‐largest public university in Bangladesh, accommodating over 33,000 students. The primary sample for this study consisted of young adults aged ≥ 18 years who were currently pursuing their undergraduate or graduate studies in any department of the university. The minimum sample size needed for this investigation was derived using the following formula:

$$n=\frac{Z_{1-\alpha_{/2}}^{2}p(1-p)}{d^{2}}.$$

On the basis of the subsequent assumptions, the sample size was determined using the single population proportion formula, with a 95% confidence level and a 5% margin of error, after 39.4% of university students in Bangladesh reported having moderate‐to‐severe depression [4]. After accounting for a 10% nonresponse rate, a design effect of 2, and a finite population correction, the minimum sample size for this study was 796. Nevertheless, a total of 900 students were invited to participate in this study during the study period. Out of these, 22 were absent, 12 were unable to attend owing to scheduling constraints, and 8 did not complete all the items of the questionnaire. This procedure led to a final sample of 858 participants, resulting in a response rate of 95%.

The data was collected using a two‐stage sampling approach. The University of Rajshahi comprises 12 faculties, each with a distinct number of enrolled students. During the initial stage, samples were distributed to each faculty based on the probability proportional to the number of students in each faculty. During the second stage, one department was randomly selected from each of the 12 faculties. The samples allotted to each faculty were subsequently distributed to the respective selected departments. Every student from the designated departments met the criteria for participation in this study. Lastly, a systematic random sample of the students' roll numbers was used to choose the participants. Each participant was provided with a comprehensive explanation of the study's objective, purpose, risks, and benefits. Written informed consent was sought from the chosen respondent before the survey was administered. Selected students were administered a pretested, structured questionnaire by two well‐trained interviewers (one male and one female) recruited from the M.Sc. students of the university. Interviewers received training on evaluating suicide risk and the emergency protocols to implement if an individual is considered to be at imminent risk of suicide. Interviewers were given the contact information of a psychiatrist for consultation in such instances. Students completed the interview voluntarily, which took place outside of the scheduled class and took 20–25 min to complete the interview. A recruited research assistant transferred the data from the paper questionnaire to an electronic data file. The ethical review committee of the Institute of Biological Sciences, University of Rajshahi, reviewed and approved the survey (Ref. 293(13)/320/IAMEBBC/IBSc).

### 2.2 Measures

#### 2.2.1 ACEs

ACEs have been assessed using various measures; we adopted the 10 items from the CDC‐developed ACE [16]. The 10‐item questionnaire was employed to evaluate the stressful and potentially traumatic experiences of participants that happened before the age of 18. The events encompassed many forms of abuse, such as physical abuse, repeated emotional abuse, and sexual abuse, as well as neglect both emotionally and physically. Additionally, the events involved parental separation or divorce, parental interpersonal violence, parental mental health and substance misuse issues, and having a household member who is incarcerated. The response options were encoded as 0 for “no” and 1 for “yes.” The ACE score is calculated by adding up the responses, resulting in a severity index that ranges from 0 to 10. This index indicates the number of adversities that an individual faces during their childhood. The ACEs in this investigation exhibited an internal consistency of 0.68. The questionnaire was translated from English to Bangla by professionals using the typical “forward–backward” process. This tool has been empirically demonstrated to possess cultural validity in Bangladesh [35].

#### 2.2.2 Anxiety and Depression Symptoms

The levels of anxiety and depression symptoms in young‐adult university students were assessed using the Generalized Anxiety Disorder 7‐item Scale (GAD‐7) [36] and the Patient Health Questionnaire‐9 (PHQ‐9) [37], respectively. Both instruments require participants to indicate the frequency of each symptom they have had in the past 2 weeks. This is done using a 4‐point Likert scale, where 0 represents “not at all” and 3 represents “almost every day.” The GAD‐7 has a potential score range of 0–21, whereas the PHQ‐9 has a potential score range of 0–27. Higher scores on both scales correspond to increased degrees of anxiety and depression, respectively. Originally developed and validated in adult primary care, both measures were self‐reported and now extend to general samples, including young adults [38, 39]. The GAD‐7 and PHQ‐9 have consistently demonstrated strong internal consistency (*α* = 0.85–0.92 for the GAD‐7 and *α* = 0.82–0.90 for the PHQ‐9) and have been found to be valid when compared to other self‐report measures of anxiety and depression [40, 41]. The optimal threshold for the GAD‐7 and PHQ‐9, which suggests a likely diagnosis of anxiety disorder or major depressive illness, differs in various sources of literature. Nevertheless, a cut‐off score of ≥ 10 on the GAD‐7 and PHQ‐9 was chosen in this investigation to classify students with moderate‐to‐severe anxiety and depression, respectively, based on the suggested literature [36, 37]. The GAD‐7 and PHQ‐9 internal consistency values in this sample were 0.81 and 0.83, respectively. All instruments (ACEs, PHQ‐9, and GAD‐7) were translated from English to Bangla by a bilingual mental health expert. The Bangla translation of those instruments was back‐translated into English by a different expert. The synthesized back translations were analyzed against the original scales to identify any discrepancies in meaning between the original and back‐translated versions.

#### 2.2.3 Control Variables

The study sample was characterized using a variety of socio‐demographic variables, including age (categorized as two groups using mean age of the respondents: 19–23 or 24–28 years), sex, academic status (undergraduate or postgraduate student), marital status (married or unmarried), religion, educational attainment of the father and mother (none or primary education, secondary education, or higher education), occupation of the father and mother, monthly income of the family, number of family members, number of family members earning income, participation in extracurricular activities, body mass index (normal, underweight, or overweight), and permanent place of residence (rural or urban).

#### 2.2.4 Statistical Analysis

Sample descriptive statistics were estimated with univariate analysis to determine the proportions and means across participants' demographic characteristics, cumulative number of ACEs, and mental health outcomes. The mean differences in ACEs among students' levels of anxiety and depression symptoms, as well as other sociodemographic variables, were examined using one‐way ANOVA or *t*‐tests, as appropriate. The chi‐squared test was used to evaluate the variations in self‐reported moderate‐to‐severe anxiety and depression symptoms among participants based on their sociodemographic variables. As follow‐ups to a significant ANOVA, Tukey honestly significant difference (HSD) post hoc multiple comparison tests were employed where appropriate. Given that the outcomes (moderate to severe anxiety: yes or no; moderate to severe depression: yes or no) of this study were binary, multivariable logistic regression analyses were employed to examine the relationship between ACEs and self‐reported moderate‐to‐severe anxiety and moderate‐to‐severe depression symptoms. The odds ratio (OR) was calculated to determine the strength of the link, and the 95% confidence interval (CI) was used to test for significance. The entire 95% CI around the OR being below 1.00 or above 1.00 indicates that the estimated OR reflects a lower risk and a higher risk, respectively. Furthermore, the estimated OR is statistically significant (*p* < 0.05) in both cases. When the 95% CI surrounding the OR includes the value of “no difference,” specifically 1.00, it indicates that the estimated OR is not statistically significant (*p* > 0.05). Statistical significance was determined as two‐sided in all analyses, with *p* values set at a 5% level of significance. Stata version 14.0 (StataCorp LP, College Station, TX) was used in conducting all the analyses.

## 3 Results

**Figure 1:**
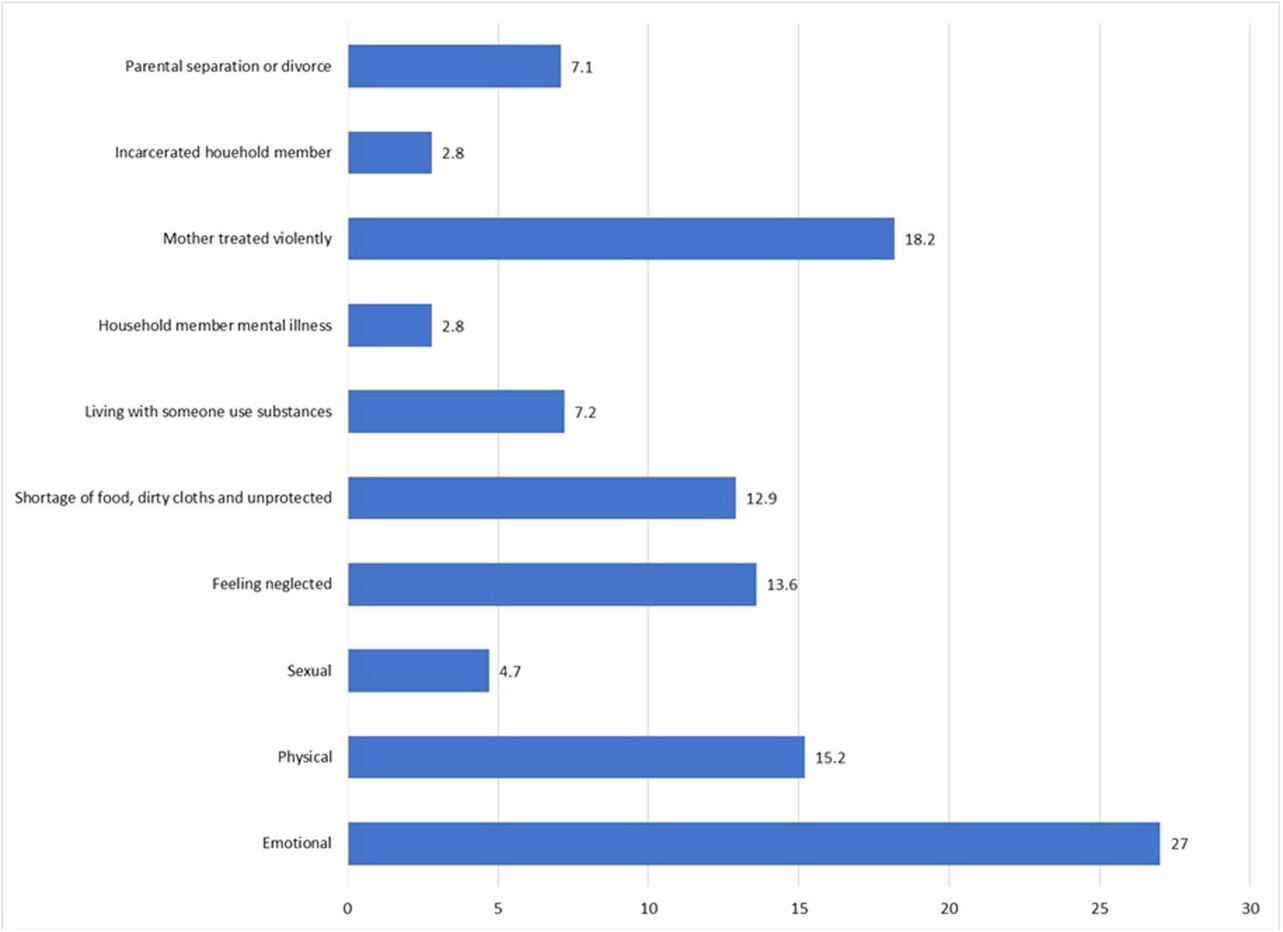
Various types of adverse childhood experiences reported by university students in Bangladesh.

**Figure 2:**
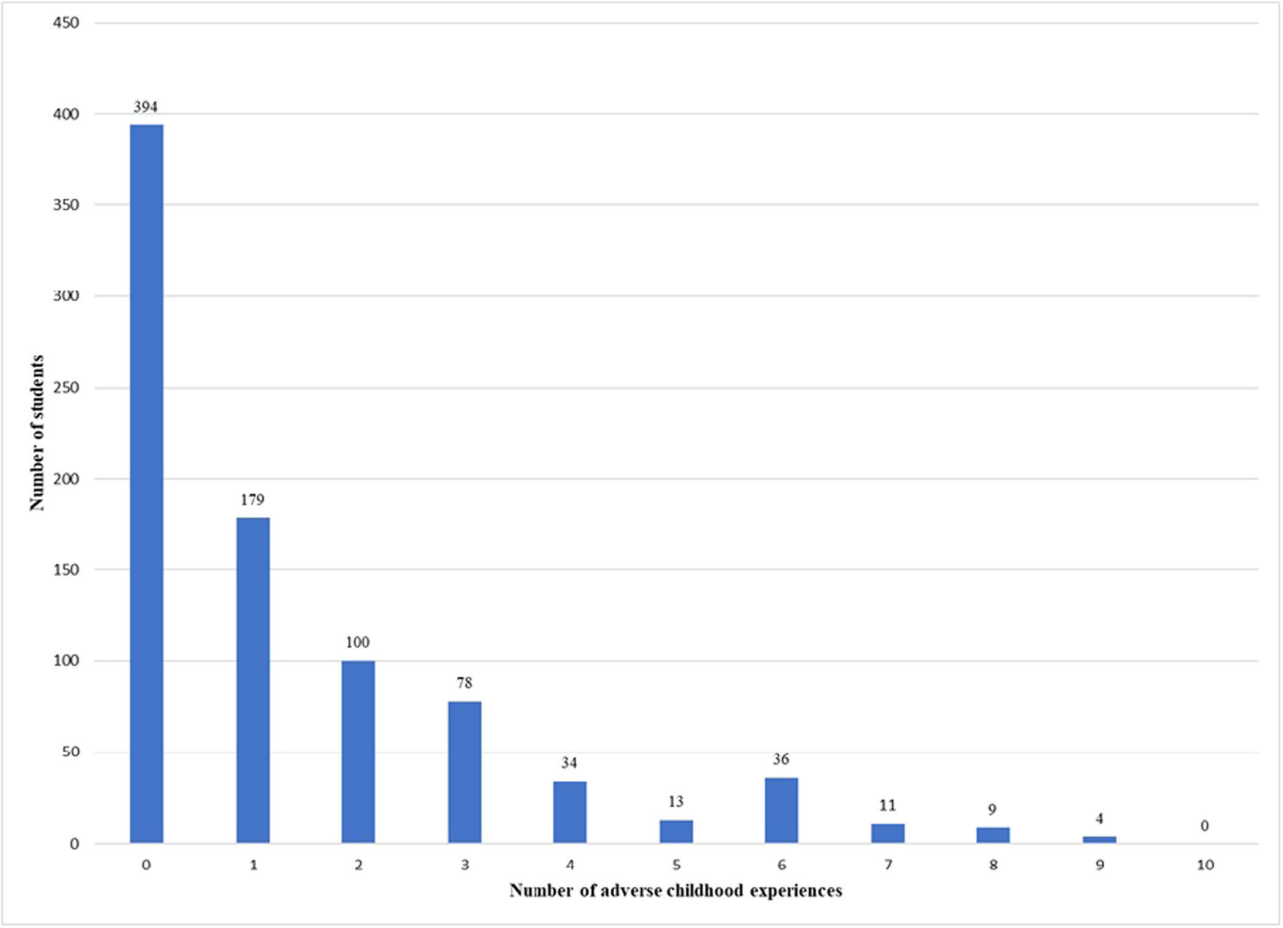
Number of students reported experience of different numbers of adverse childhood experiences (ACEs).

Table 1 displays the characteristics of the sample. The mean age of the respondents was (mean ± SD) 23.03 ± 1.79 (range: 19–28) years. The sample consists of 54.2% male respondents, and 83.1% were undergraduate students. Nearly 85% of the students were Muslims, and 90.2% were not married. Approximately half (52.6%) of the respondents had a father with a higher education, and 37.9% of respondents had a mother with a higher education. One‐fifth of the respondents' fathers were farmers, 28.2% were in government services, and 34.6% were in business or other occupations. More than half of the students (58.3%) were not engaged in any extracurricular activities, and 40.8% of the respondents were from rural backgrounds. On average, 1.42 ± 1.92 ACEs were reported by the students. The prevalence of moderate‐to‐severe anxiety and depression symptoms was 34.4% and 71.6%, respectively. Figure 1 illustrates various types of ACEs reported by the students. The predominant type of ACEs reported by students was emotional abuse (27.0%), followed by maternal violence (18.2%), physical abuse (15.2%), feelings of neglect (13.6%), and lack of food, dirty clothing, and a sense of insecurity (12.9%). Figure 2 represents the number of students reported experiencing different numbers of ACEs. It shows that 45.9% did not experience any ACE before reaching 18 years of age, while 20.9% reported experiencing one ACE, 11.7% reported two ACEs, and 0.5% reported nine ACEs.

**Table 1:** Sample characteristics (*N* = 858).

Characteristics	Number (*n*)	Percent (%) or mean (SD)
Age (Range: 19–28)		23.03 (1.76)
19–23	478	55.7
24–28	380	44.3
Sex		
Male	465	54.2
Female	393	45.8
Academic status		
Undergraduate	713	83.1
Postgraduate	145	16.9
Marital status		
Married	84	9.8
Not married	774	90.2
Religion		
Non‐Muslims	133	15.5
Muslims	725	84.5
Father's education		
No education or Primary	176	20.5
Secondary	231	26.9
Higher	451	52.6
Mother's education		
No education or Primary	253	29.5
Secondary	280	32.6
Higher	325	37.9
Father's occupation		
Farmer	185	21.6
Government service	242	28.2
Nongovernment service	134	15.6
Business or others	297	34.6
Mother's occupation		
Homemakers	675	78.7
Service or others	183	21.3
Socioeconomic status		
< 40,000 BDT	560	65.3
≥ 40,000 BDT	298	34.7
Family member		
2–4	441	51.4
≥ 5	417	48.6
Earning member in the family		
1	555	64.7
≥ 2	303	35.3
Extracurricular activities		
Not engaged	500	58.3
Engaged	358	41.7
Body mass index (BMI)		
Normal	574	66.9
Underweight	127	14.8
Overweight	157	18.3
Permanent place of residence		
Rural	508	59.2
Urban	350	40.8
Adverse childhood experiences (ACEs)	858	1.42 (1.92)
Moderate to severe anxiety	295	34.4
Moderate to severe depression	614	71.6

Abbreviations: BDT, Bangladeshi Taka; SD, Standard deviation.

Table 2 represents the mean differentials of ACEs by self‐reported anxiety and depression symptoms and other sociodemographic variables. The mean ACEs were reported to be higher among students who reported moderate‐to‐severe anxiety symptoms (1.90 ± 2.00 vs. 1.16 ± 1.83; *t*[856, *n* = 858] = −5.477, *p* < 0.001) and depression symptoms (1.51 ± 1.85 vs. 1.17 ± 2.07; *t*[856, *n* = 858] = 2.311; *p* = 0.021) than minimal anxiety and depression symptoms. Average ACEs were higher among students who were from urban areas than from rural areas (1.83 ± 2.27 vs. 1.13 ± 1.58; *t*[856, *n* = 858] = 5.301, *p* < 0.001). The experiences of adversity before age 18 were significantly higher among respondents who were female, undergraduate students, married, engaged in extracurricular activities, Muslims, or had higher socioeconomic status. The Tukey HSD post hoc multiple comparison tests were employed for significant ANOVA, and the results are presented in Table S1.

**Table 2:** Mean differentials of adverse childhood experiences by mental health outcomes and others sociodemographic variables among young adult university students (*N* = 858).

Mental health outcomes	Number of ACEs experiences Mean (SD)	*p* value (*F*/*t*‐test)
Age		*t* = −0.962, *df* = 856, *p* = 0.168
19–23	1.36 (1.71)	
24–28	1.49 (2.15)	
Sex		*t* = −3.172, *df* = 856, *p* = 0.002
Male	1.22 (1.89)	
Female	1.64 (1.93)	
Academic status		*t* = 3.499, *df* = 856, *p* = 0.001
Undergraduate	1.52 (2.01)	
Postgraduate	0.91 (1.30)	
Marital status		*t* = 6.877, *df* = 856, *p* < 0.001
Married	2.75 (2.08)	
Not married	1.27 (1.85)	
Religion		*t* = −2.082, *df* = 856, *p* = 0.038
Non‐Muslims	1.10 (1.13)	
Muslims	1.47 (2.03)	
Father's education		*F* = 6.88, *df* = 857, *p* = 0.001
No education or Primary	1.04 (1.35)	
Secondary	1.28 (1.92)	
Higher	1.63 (2.08)	
Mother's education		*F* = 7.80, *df* = 857, *p* < 0.001
No education or Primary	1.04 (1.41)	
Secondary	1.46 (2.09)	
Higher	1.67 (2.06)	
Father's occupation		*F* = 13.82, *df* = 857, *p* < 0.001
Farmer	1.16 (1.75)	
Government service	1.54 (2.15)	
Nongovernment service	2.28 (2.46)	
Business or others	1.09 (1.34)	
Mother's occupation		*t* = −3.930, *df* = 856, *p* < 0.001
Homemakers	1.28 (1.88)	
Service or others	1.90 (1.98)	
Socioeconomic status		*t* = −3.379, *df* = 856, *p* = 0.001
< 40,000 BDT	1.25 (1.72)	
≥ 40,000 BDT	1.72 (2.22)	
Family member		*t* = −0.444, *df* = 856, *p* = 0.657
2–4	1.38 (1.89)	
≥ 5	1.44 (1.95)	
Earning member in the family		*t* = 1.379, *df* = 856, *p* = 0.168
1	1.48 (1.80)	
≥ 2	1.29 (2.11)	
Extracurricular activities		*t* = −3.042, *df* = 856, *p* = 0.002
Not engaged	1.25 (1.75)	
Engaged	1.65 (2.11)	
Body mass index (BMI)		*F* = 1.77, *df* = 856, *p* = 0.171
Normal	1.34 (1.90)	
Underweight	1.69 (2.07)	
Overweight	1.46 (1.85)	
Permanent place of residence		*t* = −5.301, *df* = 856, *p* < 0.001
Rural	1.13 (1.58)	
Urban	1.83 (2.27)	
Anxiety		*t* = −5.477, *df* = 856, *p* < 0.001
Minimal	1.16 (1.83)	
Moderate to severe	1.90 (2.00)	
Depression		*t* = −2.311, *df* = 856, *p* = 0.021
Minimal	1.17 (2.07)	
Moderate to severe	1.51 (1.85)	

Abbreviations: BDT, Bangladeshi Taka; SD, Standard deviation.

Table 3 represents the bivariate relationship between students self‐reported moderate‐to‐severe anxiety and depression symptoms and different sociodemographic variables. Female students were more likely to have moderate‐to‐severe anxiety symptoms than male respondents (40% vs. 29.7%; *χ* ^2^[1, *n* = 858] = 9.961, *p* = 0.002). While moderate‐to‐severe anxiety symptoms were higher among undergraduate students (36% vs. 26.2%; *χ* ^2^[1, *n* = 858] = 5.169, *p* = 0.023), moderate‐to‐severe depression symptoms was reported to be higher among postgraduate students (79.3% vs. 70%; *χ* ^2^[1, *n* = 858] = 5.148, *p* = 0.023). Moderate‐to‐severe anxiety symptoms (*p* < 0.001) and depression symptoms (*p* < 0.001) were significantly associated with a father's education. The father's occupation was also significantly associated with moderate‐to‐severe anxiety symptoms (*p* < 0.001) and depression symptoms (*p* = 0.017). Students who were engaged in any extracurricular activities were less likely to report moderate‐to‐severe depression symptoms (65.9% vs. 75.6%; *χ* ^2^[1, *n* = 858] = 9.602, *p* = 0.002).

**Table 3:** Bivariate relationship between students self‐reported mental health outcomes and different socio‐demographic and health‐related variables (*N* = 858).

Characteristics	Moderate to severe anxiety, *n* (%)	*p*‐value (*χ* ^2^‐test)	Moderate to severe depression, *n* (%)	*p*‐value (*χ* ^2^‐test)
Age		*χ* ^2^ = 1.130, *df* = 1, *p* = 0.288		*χ* ^2^ = 8.437, *df* = 1, *p* = 0.004
19–23	157 (32.8)		323 (67.6)	
24–28	138 (36.3)		291 (76.6)	
Sex		*χ* ^2^ = 9.961, *df* = 1, *p* = 0.002		*χ* ^2^ = 0.898, *df* = 1, *p* = 0.343
Male	138 (29.7)		339 (72.9)	
Female	157 (40.0)		275 (70)	
Academic status		*χ* ^2^ = 5.169, *df* = 1, *p* = 0.023		*χ* ^2^ = 5.148, *df* = 1, *p* = 0.023
Undergraduate	257 (36.0)		499 (70.0)	
Postgraduate	38 (26.2)		115 (79.3)	
Marital status		*χ* ^2^ = 3.633, *df* = 1, *p* = 0.057		*χ* ^2^ = 0.001, *df* = 1, *p* = 0.977
Married	21 (25.0)		60 (71.4)	
Not married	274 (35.4)		554 (71.6)	
Religion		*χ* ^2^ = 8.033, *df* = 1, *p* = 0.005		*χ* ^2^ = 0.348, *df* = 1, *p* = 0.555
Non‐Muslims	60 (45.1)		98 (73.7)	
Muslims	235 (32.4)		516 (71.2)	
Father's education		*χ* ^2^ = 16.202, *df* = 2, *p* < 0.001		*χ* ^2^ = 33.770, *df* = 2, *p* = < 0.001
No education or primary	54 (30.7)		154 (87.5)	
Secondary	59 (25.5)		142 (61.5)	
Higher	182 (40.3)		318 (70.5)	
Mother's education		*χ* ^2^ = 3.184, *df* = 2, *p* = 0.204		*χ* ^2^ = 3.674, *df* = 2, *p* = 0.159
No education or primary	77 (30.4)		189 (74.7)	
Secondary	96 (34.3)		189 (67.5)	
Higher	122 (37.5)		236 (72.6)	
Father's occupation		*χ* ^2^ = 444.481, *df* = 3, *p* < 0.001		*χ* ^2^ = 13.561, *df* = 3, *p* = 0.004
Farmer	47 (25.4)		134 (72.4)	
Government service	63 (26.0)		167 (69.0)	
Nongovernment service	39 (29.1)		82 (61.2)	
Business or others	146 (49.2)		231 (77.8)	
Mother's occupation		*χ* ^2^ = 1.543, *df* = 1, *p* = 0.214		*χ* ^2^ = 5.731, *df* = 1, *p* = 0.017
Homemakers	225 (33.3)		496 (73.5)	
Service or others	70 (38.2)		118 (64.5)	
Socioeconomic status		*χ* ^2^ = 0.005, *df* = 1, *p* = 0.945		*χ* ^2^ = 0.457, *df* = 1, *p* = 0.499
< 40,000 BDT	193 (34.5)		405 (72.3)	
≥ 40,000 BDT	102 (34.2)		209 (70.1)	
Family member		*χ* ^2^ = 0.003, *df* = 1, *p* = 0.957		*χ* ^2^ = 3.078, *df* = 1, *p* = 0.079
2–4	152 (34.5)		304 (68.9)	
≥ 5	143 (34.3)		310 (74.3)	
Earning member in the family		*χ* ^2^ = 3.927, *df* = 1, *p* = 0.048		*χ* ^2^ = 0.435, *df* = 1, *p* = 0.509
1	204 (36.8)		393 (70.8)	
≥ 2	91 (30.0)		221 (72.9)	
Extracurricular activities		*χ* ^2^ = 0.204, *df* = 1, *p* = 0.653		*χ* ^2^ = 9.602, *df* = 1, *p* = 0.002
Not engaged	175 (35.0)		378 (75.6)	
Engaged	120 (33.5)		236 (65.9)	
BMI		*χ* ^2^ = 7.261, *df* = 2, *p* = 0.027		*χ* ^2^ = 8.395, *df* = 2, *p* = 0.015
Normal	181 (31.5)		393 (68.5)	
Underweight	55 (43.3)		97 (76.4)	
Overweight	59 (37.6)		124 (79.0)	
Permanent place of residence		*χ* ^2^ = 25.698, *df* = 1, p ≤ 0.001		*χ* ^2^ = 0.296, *df* = 1, *p* = 0.586
Rural	140 (27.6)		360 (70.9)	
Urban	155 (44.3)		254 (72.6)	

Abbreviations: BDT, Bangladeshi Taka; BMI, body mass index.

Results from the bivariate and multivariable logistic regression analyses are presented in Table 4. Both bivariate and multivariate results from Table 4 show that an increase in the ACE score increases the odds of experiencing moderate‐to‐severe anxiety and depression symptoms. The odds of moderate‐to‐severe anxiety symptoms (unadjusted odds ratio [UOR]: 1.21; 95% CI: 1.13–1.30) and depression symptoms (UOR: 1.10; 95% CI: 1.01–1.20) were higher with experiencing a higher number of ACEs. Table 4 also indicates that a one‐item increase in the ACE score increased the odds of experiencing moderate‐to‐severe anxiety symptoms by 27% (adjusted odds ratio [AOR]: 1.27; 95% CI: 1.16–1.38) and moderate‐to‐severe depression symptoms by 19% (AOR: 1.19; 95% CI: 1.08–1.31) after controlling for sociodemographic variables. Muslims were less likely to report moderate‐to‐severe anxiety symptoms (AOR: 0.48; 95% CI: 0.31–0.76) and depression symptoms (AOR: 0.71; 95% CI: 0.55–0.95) than their non‐Muslim counterparts. Students whose family has more than one earning member were 0.57 and 0.79 times less likely to report anxiety symptoms (AOR: 0.57; 95% CI: 0.37–0.87) and depression symptoms (AOR: 0.79; 95% CI: 0.59–0.98). Engagement in extracurricular activities was also found to be associated with moderate‐to‐severe depression symptoms with lower odds (AOR: 0.64; 95% CI: 0.45–0.92). Students from urban backgrounds were more likely to report experiencing moderate‐to‐severe anxiety symptoms (AOR: 1.93; 95% CI: 1.25–2.68) than their rural counterparts.

**Table 4:** Multivariable logistic regression analyses of the relationship between adverse childhood experiences and students' self‐reported mental health outcomes.

Characteristics	Moderate to severe anxiety		Moderate to severe depression AOR (95% CI)	
	UOR (95% CI)	AOR (95% CI)	UOR (95% CI)	AOR (95% CI)
Experience of ACEs	1.21*** (1.13–1.30)	1.27*** (1.16–1.38)	1.10* (1.01–1.20)	1.19*** (1.08–1.31)
Age				
19–23		1		1
24–28		1.21 (0.83–1.75)		1.36 (0.92–1.99)
Sex				
Male		1		1
Female		1.29 (0.90–1.85)		0.82 (0.55–1.20)
Academic status				
Undergraduate		1		
Postgraduate		0.58* (0.36–0.95)		1.36 (0.81–2.29)
Marital status				
Married		1		1
Not married		1.87 (0.96–3.67)		0.70 (0.38–1.28)
Religion				
Non‐Muslims		1		1
Muslims		0.48** (0.31–0.76)		0.71* (0.55–0.95)
Father's education				
No education or Primary		1		1
Secondary		0.36*** (0.20–0.63)		0.13*** (0.07–0.25)
Higher		1.42 (0.77–2.62)		0.20*** (0.10–0.41)
Mother's education				
No education or		1		1
Secondary		1.92 (0.60–1.73)		1.28 (0.75–2.17)
Higher		0.77 (0.41–1.46)		2.73** (1.40–5.30)
Father's occupation				
Farmer		1		1
Government service		0.40** (0.21–0.77)		0.84 (0.44–1.58)
Nongovernment service		0.41* (0.20–0.81)		0.44* (0.22–0.87)
Business or others		2.49*** (1.49–4.17)		1.99* (1.15–3.44)
Mother's occupation				
Homemakers		1		1
Service or others		1.06 (0.64–1.79)		0.41*** (0.25–0.67)
Socioeconomic status				
< 40,000 BDT		1		1
≥ 40,000 BDT		1.16 (0.74–1.80)		1.59* (1.03–2.25)
Family member				
2–4		1		1
≥ 5		1.41 (0.97–2.06)		1.52* (1.03–2.25)
Earning member in the family				
1		1		1
≥ 2		0.57* (0.37–0.87)		0.79* (0.59–0.98)
Extracurricular activities				
Not engaged		1		1
Engaged		1.11 (0.79–1.57)		0.64* (0.45–0.92)
BMI				
Normal		1		1
Underweight		2.34** (1.41–3.90)		2.24* (1.33–3.78)
Overweight		1.31 (0.86–2.00)		1.66** (1.05–2.63)
Permanent place of residence				
Rural		1		1
Urban		1.93** (1.25–2.68)		1.09 (0.73–1.65)
LR *χ* ^2^(*df*)	27.77 (1)***	174.12 (21)***	5.59 (1)*	125.12 (21)***
Pseudo *R* ^2^	0.025	0.158	0.006	0.122

Abbreviations: ACE, adverse childhood experience; AOR, adjusted odds ratio; BMI, body mass index; 95% CI, 95% confidence interval; UOR, unadjusted odds ratio.

* *p* < 0.05

** *p* < 0.01

*** *p* < 0.001.

## 4 Discussion

The current study sought to evaluate the relationship between ACEs and negative mental health outcomes, particularly anxiety and depression symptoms, in a sample of young adult university students in Bangladesh. This study contributes to the increasing body of literature on ACEs by demonstrating that more than half of the young adult students had been exposed to one or more ACEs. Our research also revealed that young adult students frequently experience adverse mental health outcomes, which aligns with past international studies, including those conducted in Bangladesh [25, 28, 42, 43]. We found higher odds of reporting adverse mental health outcomes among students who were exposed to ACEs. The higher prevalence of ACEs among university students and their probable correlation with mental health problems underscore the significance of prioritizing safeguarding children in this country.

The prevalence of ACEs among young adults in this study is consistent with other studies conducted across the globe, where they reported the prevalence of ACEs among university students was more than 50% [20, 26, 28]. Nevertheless, our reported prevalence of ACEs is comparatively lower than the findings of earlier research in Bangladesh, where the reported prevalence of psychological abuse was 97% and physical abuse was 99% [22, 23]. The observed variations may be attributed to their utilization of samples consisting of either adolescents or the elderly, as well as the inclusion of samples from rural areas. The inconsistencies in the findings could also possibly be attributed to the discrepancies in the assessment methodology of ACEs. Nonetheless, our findings indicated that children in Bangladesh are relatively frequently exposed to adversities in their households. The risk of ACE exposure in this cultural group may be increased by deeply rooted values and beliefs, such as acceptance of physical punishment, normalization of family violence, and patriarchal norms, which may expose children to more direct and indirect forms of violence at home [28].

In line with a large body of research [25, 26, 27, 28, 29, 30], our findings indicate a significant relationship between exposure to ACEs and the development of anxiety and depression symptoms during adulthood. The transition from adolescence to young adulthood is a unique phase in one's life that is characterized by semi‐autonomous exploration and major milestones such as leaving the family home, beginning university or work, getting married, and becoming a parent [44]. A longitudinal study conducted on US college students revealed a significant decrease in their psychological well‐being upon entering college [45]. This decline may be attributed to the possibility that individuals who have experienced traumatic events during childhood are more susceptible to experiencing lower psychological well‐being during their adjustment to college. Furthermore, global research [46], including those carried out in Bangladesh [47, 48, 49], has indicated that during the period of emerging adulthood, there is a notable rise in the prevalence of risky behaviors such as substance abuse, engaging in risky sexual activities, and reckless driving. Young adults who experience adversities in childhood exhibit a greater likelihood of exhibiting these risky behaviors [50], which are also strongly associated with developing anxiety and depression symptoms in young adults [51]. This phenomenon could explain why individuals who have experienced ACE are more prone to developing anxiety and depression symptoms.

The increased prevalence of anxiety and depression in individuals who have experienced ACEs may be influenced by a multifaceted interplay of behavioral, demographic, and biological factors. For example, ACEs are linked to increased inflammation in adulthood, specifically higher levels of C‐reactive protein [52], which has been identified as a mediator of the connection between ACEs and depression [53]. Moreover, when early ACE‐induced mood changes occur, they activate a biological stress response, which affects the HPA axis. This stimulation causes the adrenal cortex to release cortisol at consistently elevated levels for an extended period of time. Consequently, such a simulation puts the person at a higher likelihood of developing depression and anxiety disorders [54].

There are a few limitations to consider when evaluating the results. This study is based on a single university student's young adult sample; thus, the conclusions cannot be generalized to all Bangladeshi young adult people. Nonetheless, the current findings are noteworthy, given the paucity of available information on this population cohort. In accordance with the majority of surveys, the exposure and outcome variables were collected through self‐report. Social desirability bias is likely to affect the accuracy of self‐reported ACE history. While this is a typical method for evaluating ACEs, it is important to acknowledge that the present mental health condition may have influenced the disclosure of ACEs [55]. In addition, underreporting is another possible source of bias. For example, ACEs tend to be underreported in adulthood. Due to either intentional non‐reporting or infantile amnesia, up to one‐third of actual occurrences of child abuse remain unreported in adulthood [56]. Despite reports of limited agreement between retrospective and prospective measures of ACEs [57], both have been linked to unfavorable adult health outcomes [55]. In fact, a recent meta‐analysis showed no variations in the associations between mental health disorders and retrospective and prospective ACE measures [58]. A further limitation is that the reliability of the ACE items presented in this study is inadequate (Cronbach *α* = 0.68). Nonetheless, several researchers have indicated that reliability coefficients ranging from 0.60 to 0.70 may be satisfactory and useful for research instruments [59, 60]. Furthermore, although we utilized widely recognized and validated screening tools to assess mental health problems, it is important to note that the self‐reported symptoms may not necessarily correspond with a formal clinical diagnosis.

Notwithstanding, the findings of the study can offer basic information and insights for stakeholders in academia, public health, and mental health, as well as health practitioners and legislators, to improve the well‐being of young adult students in the context of ACEs. Given that emerging adulthood is a critical period for the onset of various mental disorders [1], an examination of university students could uncover the earlier health impacts of ACEs, potentially illuminating effective strategies for intervention. It is imperative for policymakers in Bangladesh to confront the underlying factors contributing to ACEs. Collaborative efforts among all related stakeholders are crucial to enhance awareness regarding the significance of preventing such adversities within families. Such goals can be achieved through focused interventions, educational initiatives, public health campaigns, better access to mental health services, counseling, and necessary legal reforms. These measures aim to foster a safer and more supportive environment for children, ultimately promoting better mental health outcomes in adulthood. The university administration should take proactive steps to share and engage in discussions about the findings of this study with both students and staff. This approach could enhance awareness regarding the challenges of childhood adversities and their associated mental health outcomes within the institution. Staff training focused on trauma‐informed care and effective educational practices could be initiated that aid in recognizing students who require support and offers them essential assistance.

## 5 Conclusion

More than half of young adult university students in Bangladesh (464 out of 858) reported having had at least one ACE, and it has been found that ACE exposure is associated with the development of symptoms of anxiety and depression. Gaining insight into these relationships can aid in reducing the effects of ACEs and averting detrimental health outcomes, such as anxiety and depression. The findings strongly indicate the necessity of conducting ACE screenings among students to identify those who are at a heightened risk of experiencing deteriorating mental health during their time in university. When creating mental illness prevention initiatives, policymakers and university authorities should take ACEs into account.

## Author Contributions

**Shamima Akter:** conceptualization, data curation, formal analysis. **Raufun Hasan Arnob:** software, formal analysis, data curation. **Md. Ashik Ulla Ashik:** writing – original draft, formal analysis. **Md. Mosfequr Rahman:** supervision, writing – review and editing, methodology.

## Conflicts of Interest

The authors declare no conflicts of interest.

## Transparency Statement

The lead author, Md. Mosfequr Rahman affirms that this manuscript is an honest, accurate, and transparent account of the study being reported; that no important aspects of the study have been omitted; and that any discrepancies from the study as planned (and, if relevant, registered) have been explained.

## Supporting information

Supplement Tables.

## Data Availability

The data set is available upon request. Interested researchers should contact Md. Mosfequr Rahman at mosfeque@ru.ac.bd for data requests.
