# Supplementary material for: Exposure to Adverse Childhood Experiences and Mental Health Issues in a Young‐Adult Sample of University Students in Bangladesh: A Cross‐Sectional Study
Source: Health Sci Rep. 2025 Apr 18;8(4):e70712. doi: 10.1002/hsr2.70712 (PMC12007430; doi:10.1002/hsr2.70712)
Supplement: Supplementary file 1 — Supplement Tables. [file HSR2-8-e70712-s001.DOCX]

**Supplementary Table 1** Results of Tukey Honestly Significant Difference (HSD) test for multiple comparisons for mean differences of the adverse childhood experiences (ACEs) across different study variables

| **Characteristics** | **Group vs Group** | **Group means** | | **Mean difference** | **HSD-test** |
| --- | --- | --- | --- | --- | --- |
| **Father’s education** | No education or primary vs. secondary | 1.0398 | 1.2814 | 0.2416 | 1.9829 |
|  | No education or primary vs. higher | 1.0398 | 1.6319 | 0.5922 | 4.8598* |
|  | Secondary vs. higher | 1.2814 | 1.6319 | 0.3505 | 2.8769 |
| **Mother’s education** | No education or primary vs. secondary | 1.0435 | 1.4571 | 0.4137 | 3.6500* |
|  | No education or primary vs. higher | 1.0435 | 1.6708 | 0.6273 | 5.5350* |
|  | Secondary vs. higher | 1.4571 | 1.6708 | 0.2136 | 1.8850 |
| **Father’s occupation** | Farmer vs. 2^nd^ Govt. service | 1.1568 | 1.5372 | 0.3804 | 2.8361 |
|  | Farmer vs. Non-govt. service | 1.1568 | 2.2761 | 1.1194 | 8.3446* |
|  | Farmer vs. business or others | 1.1568 | 1.0909 | 0.0658 | 0.4909 |
|  | Govt. service vs. Non-govt. service | 1.5372 | 1.2761 | 0.7389 | 5.5086* |
|  | Govt. service vs. business or others | 1.5372 | 1.0909 | 0.4463 | 3.3269 |
|  | Non-govt. service vs. business or others | 1.2761 | 1.0909 | 1.1852 | 8.8355* |

**p*<0.05
